# Multi-Layer IoT Security Framework for Ambient Intelligence Environments

**DOI:** 10.3390/s19184038

**Published:** 2019-09-19

**Authors:** Ion Bica, Bogdan-Cosmin Chifor, Ștefan-Ciprian Arseni, Ioana Matei

**Affiliations:** Faculty of Information Systems and Cyber Security, “Ferdinand I” Military Technical Academy, 050141 Bucharest, Romania; bogdan.chifor@mta.ro (B.-C.C.); ioana.matei@mta.ro (I.M.)

**Keywords:** IoT, security framework, remote attestation, packet filtering, trust management

## Abstract

Ambient intelligence is a new paradigm in the Internet of Things (IoT) world that brings smartness to living environments to make them more sensitive; adaptive; and personalized to human needs. A critical area where ambient intelligence can be used is health and social care; where it can improve and sustain the quality of life without increasing financial costs. The adoption of this new paradigm for health and social care largely depends on the technology deployed (sensors and wireless networks), the software used for decision-making and the security, privacy and reliability of the information. IoT sensors and wearables collect sensitive data and must respond in a near real-time manner to input changes. An IoT security framework is meant to offer the versatility and modularization needed to sustain such applications. Our framework was designed to easily integrate with different health and social care applications, separating security tasks from functional ones and being designed with independent modules for each layer (Cloud, gateway and IoT device), that offer functionalities relative to that layer.

## 1. Introduction

Even if it is a relatively new concept, the Internet of Things (IoT) opens perspectives that can change the way we interact, not only with each other but also with the environment surrounding us. IoT is an emerging field that has received a lot of attention from many industries, allowing the development of applications for automotive, transport, telecommunication or ambient intelligence. Beyond the tempting facilities that IoT solutions offer, the innovation aspects are more enlightening in ambient intelligence. In this area, IoT applications address optimization, efficiency or ease-of-use issues, and devices work in order to support people in carrying out their everyday life activities. IoT devices can provide an approach that enables older people or people with disabilities to live in a more autonomous and personalized ambient, increasing their independence and enabling them to solve certain routine procedures on their own, positively influencing the quality of their living standards.

In ambient intelligence applications, data from sensors provides important information about the current state of the environment, and in order to make the right decisions, data must be accurate. But as in any IoT system, there may appear irregularities in data accuracy. False positives, abnormal values or misinterpretations can occur due to sensors nature, hardware faults, compromised nodes, electromagnetic interference, etc. Therefore, to prevent these problems, it is necessary to implement a solution capable of filtering out abnormal measurements so that differentiation between node defects and the real situations that require the immediate involvement of specialized personnel, can be made.

There are ambient intelligence applications that require high security measures due to the critical nature of the sensed information, especially when taking into consideration the healthcare context (e.g., medical information). Traditional security methods are difficult to implement in this case because an IoT application usually consists of different interconnected hardware (resource constrained) and software components from multiple vendors which raise many issues. Considering this context, there are many particularities that need to be addressed by a security solution: a large number of devices, each with its unique capability and purpose, ad-hoc connections, increased difficulty in managing the entire system and different methods through which devices interact with consumers (humans or other systems). In health and social care applications that process sensitive data, security and privacy measures must be implemented without affecting service efficiency. Thus, a feasible security solution needs to implement protection mechanisms at all layers of the IoT system (e.g., devices, gateways, Cloud), while still allowing uninterrupted and transparent communications between these layers.

To develop an ambient intelligence application, one can use an existing IoT platform, such as AWS IoT from Amazon, ARM Bed from ARM and other partners, Azure IoT Suite from Microsoft, Brillo/Weave from Google, Calvin from Ericsson, HomeKit from Apple, Kura from Eclipse, or SmartThings from Samsung. These platforms allow rapid application development and support a broad range of sensors and wearables. In addition, each platform encapsulates standard security mechanisms like authentication, authorization, access control, and secure communication, as presented in [1]. However, important security challenges required by critical IoT applications, including health and social care ones, are not handled by these platforms. Data anomaly detection, remote attestation or packet filtering are issues that need to be addressed to be able to mitigate complex attacks.

In this paper, we propose a security framework that tackles these challenges, bringing improvements to ambient intelligence applications, with emphasis on the health and social care monitoring. To achieve this, the proposed framework consists of interconnected modules that are integrated at each of the main layers of an IoT system: Cloud, gateway, and device. The framework is built on a customized decentralized architecture, empowering middle-layer devices, such as gateways, while having a central point of management through a Cloud platform. The framework’s key components are presented in the remaining sections of the paper, which has the following structure. Section 2 presents the related work being done in this domain. In Section 3 we describe the proposed security framework, while in Section 4 we present the tests and analyses we have done on the framework modules. Section 5 ends the paper with conclusions and future research directions.

## 2. Related Work

The proposal made in [2] consists of a layered architecture of an IoT system and aspects of hardware and software design. At the same time, several areas of application of the proposed model such as smart cities, agriculture or healthcare, have been outlined. In [3], the authors have studied the state-of-the-art for IoT and pointed out that there are two important issues to be taken into account when designing an IoT system: the privacy of the people and the confidentiality of the services and processes. In order to prevent these problems, it is necessary to design an architecture tailored to the purpose of the project, then to adopt a security framework that meets the requirements of privacy and confidentiality.

A complex IoT system is characterized by a large number of heterogeneous devices that need to be managed and have their data properly collected and processed. This means that the data must be analyzed and filtered in order to identify the compromised nodes and to detect anomalies of the recorded data. Anomaly detection is a resource-consuming process, meaning that it must be made at the gateway or cloud layer given that the nodes have a low power processor and are based on low power consumption. There are many ways proposed by researchers to detect data anomalies: machine learning, recurrent calculation, statistical calculation, decision trees [4], but catching the abnormality data becomes even more difficult when there is a chance of communication errors or some intentional injection of malicious data by an attacker.

A considerable effort has been made by the research and industry communities, in presenting generic IoT frameworks, such as Internet of Things Architecture (IoT-A) [5] or Core Platform of the Future Internet (FiWARE) [6] for solving the above mentioned problems. Whilst the second framework, FiWARE, implements a “security-by-design” paradigm with generic components (such as monitoring, identity, and confidentiality management, context security or secure storage), the first one, IoT-A, proposes a trust model that considers the following security aspects:

- Trust domains—an IoT device can collect data from different domains;
- Trust evaluation mechanisms—a device reputation is calculated based on direct observations or by querying neighboring IoT devices/gateways;
- Behavior analysis policies—shape the interaction between two IoT devices, based on their reputation levels;
- Trust anchor—an entity with the highest reputation, trusted by all devices in the IoT system;
- Trust federalization—delegate the trust evaluation mechanism to another entity, in order to ensure interoperability.

Another security framework designed for unattended environments to control nodes that have access to the network is presented in [7]. The access control is based on administrative approval in order to ensure compliance with defined security rules. In a network intended to monitor the functions of the human body and the environment, the information that transits the network is life-critical. So there is a need to deploy security mechanisms for preventing malicious attacks. The basis for these is to define the standards and protocols used to enhance data security. A significant improvement has been seen in the IEEE 802.15.4 and is broadly explained in [8].

Regardless of what framework an IoT system is built upon, the large number of integrated devices transforms the notions of trust in collected information and the reputation of IoT devices into two key concepts [9]. These two concepts are also directly linked: depending on the devices’ reputation, data collected from it is tagged with a specific level of trust by a user. In health and social care applications, data cannot be assessed anymore as simple environmental data, without any reference to a person’s habits; instead, it needs to be split into different categories, according to their origin and future use (e.g., data collected from Personal Area Networks (PAN) [10] or Body Area Networks (BAN) [11] needs either to be processed locally or to be anonymized before being sent in Cloud and the users need to have full control of it, while environmental data can be sent and processed remotely without the need of applying the same level of filtering as in the previous case). Thus, in these types of applications, trust is not only a characteristic of data read from sensors and their reputation, but it becomes also a requirement for the functioning of the entire IoT environment (users need not only to trust received data but also to trust the way the system manipulates that data, before and after it is being presented to them). This second “level” of trust can be achieved through security mechanisms that ensure privacy and protection of data throughout the entire lifecycle of that data. Returning to the link between the first “level” of trust and reputation of nodes, as stated at the beginning of this paragraph, the notion of reputation can be used to define the resulting value of the process of assessing IoT devices based on their behavior features, such as: transaction history, reliability of established communication links or quality of sent data. Each one of these features impacts the way an IoT device (a node in the IoT network) is perceived by other nodes. An IoT device trustworthiness impacts directly the connections with other devices: data published by a device with a low reputation score has a low probability of being consumed. Thus, a malicious node introduced in the IoT system would be detected through its behavior, no matter when an attack is launched. Considering this capability, many models for assessing the reputation level of a node have been proposed, based on different mathematics, physics or other aspects, such as Bayesian [12], subjective logic [13], entropy [14,15] or biological elements [16,17]. Even though the concept of a reputation assessment model can be for a variety of domains, authors in [18,19] have highlighted five essential stages that a reputation-based architecture needs to have built-in:

- Data collection—query the node or its neighbors for behavioral information;
- Scoring and reputation level assessment—reputation score of a node is calculated based on a reputation assessment model (one of those described before or a similar one);
- Nodes selection—valid nodes can consider data rerouting, based on updated reputation scores of their neighboring nodes;
- Transaction execution—after a data transfer is finished, a transaction is considered executed, then the user can give it a score;
- Updating the reputation level of a node—based on the users’ score, nodes can be “rewarded” or “punished”, affecting their reputation.
- Furthermore, authors in [20] emphasize several characteristics that must be considered before integrating security features based on reputation in an IoT system architecture:
- Users’ partiality in scoring nodes;
- Possible transitivity of reputation across a trust chain;
- Initial context in which primary trust relations are defined (based on given reputation scores).

Extending the applications commonly found in an IoT system, the authors in [21] propose the deployment of a reputation solution for evaluating data presented by devices in a participatory sensing environment. Given the dynamic nature of this sensing environment, the adaptivity of a reputation-based evaluation architecture is another advantage and a desired characteristic.

Although these reputation-based security mechanisms can bring improvements in the overall security of an IoT system, they are not protected against some classic attack methods that can be adapted to work in a reputation-assessing architecture. Such examples of attacks are mentioned in [20]. To counter these possible problems the IoT architecture needs to have regular security mechanisms integrated, such as Transport Layer Security (TLS) / Datagram Transport Layer Security (DTLS) communications, authentication and Access Control Lists (ACL). Another issue with reputation-based security mechanisms is the overhead in power consumption, given the additional computations needed to evaluate the reputation for each node. One direction for assessing the impact of this issue in the overall performance of an IoT system is the modeling of energy consumption in sensor networks using stochastic Petri networks (SPNs), as presented in [22]. This approach can give a prediction of power consumption, thus enabling gateways to adjust the computations required by the reputation evaluation algorithm, at different levels of battery power.

## 3. Proposed IoT Security Framework

The design of a security framework for ambient intelligence environments should be based on a modular architecture that allows scalability, given that IoT networks are widely adopted today. In the beginning, a typical context of ambient intelligence was homes, and over time it expanded to workspaces, public spaces and hospital environments. The proposed framework has a multi-level structure and includes at each level (node, gateway, and Cloud) a component mandated to monitor and act independently if the module is prone to attacks. The architecture presented in the following subsections is based on the classic centralized model but introduces decentralized architecture elements by using the gateway as a key element. This is because all nodes have a single communication link which implies that any connection with another node will be monitored and managed by the gateway, thus allowing the local control of the resources, with the Cloud module acting as a supervisor.

An important feature that must be provided by an IoT security framework in the ambient intelligence applications consists of a secure infrastructure that allows the data transmission from the end-point sensors to the cloud services. Also, an IoT security framework must offer the infrastructure for additional services, like sensor anomaly detection which is critical in the healthcare context. Our proposed framework offers the support for running an anomaly detection algorithm on the gateway side, in order to detect sensor data anomalies in a real time manner, thus satisfying the requirements of a healthcare application. Moreover, the gateway element in our system processes requests for two data categories in order to address various ambient environment applications: critical healthcare data which must be processed immediately and environment context data which is not critical, but which can lead to better knowledge about the persons’ health state. Given the importance of the gateway in our system, we implemented a low cost Denial of Service (DoS) mitigation solution which assures another important requirement of an ambient intelligence environment application: the system availability.

**Figure 1:**
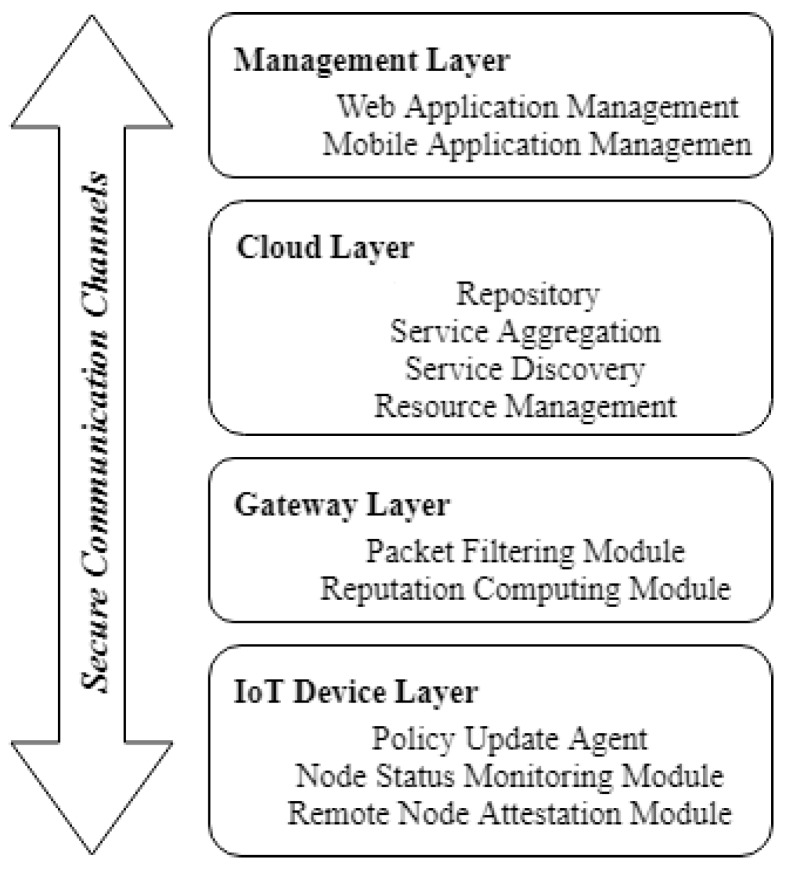
The architecture of the proposed security framework.

As presented in Figure 1, the proposed solution integrates various security components at all levels of the IoT system. The interaction of different security modules is ensured by establishing a trust relationship based on the authentication mechanism. The IoT security framework allows establishing these trust relationships using X.509 digital certificates, asymmetric keys or pre-shared symmetric keys, depending on the constraints of the devices involved. In addition to this, the solution includes an anomaly detection module by which node behavior is observed and quantified at the gateway level to create a more trustworthy system.

### 3.1. Cloud Layer

As we mentioned before, the feature that links our proposed security framework with other centralized IoT systems is the Cloud module that acts as an access point for users to connect to different services and as a management module for the underlying IoT system, but with reduced capabilities. The Proposed Cloud Platform (PCP) was not designed as a replacement for the Existing Cloud Platforms (ECP), but as a security “complement” for them, adding additional features to enforce control over devices (behavior and data). Data is exchanged between these two Cloud platforms by means of APIs exposed by the ECP. In this manner, we ensure that data coming from gateways is filtered so that the ECP does not receive corrupted information and that users obtain the correct results when accessing services. Thus, the first functionality from the reduced set that the PCP implements is to be an element of overseeing and enforcing specific security rules and policies, meant to limit the infusion of malicious data due to a rogue gateway. Aside from filtering traffic flows, the PCP acts also as a service discovery module for the gateway and devices layers, by aggregating services available on different ECPs. As a mention, the services that are aggregated by the PCP are services that are allowed by the administrators of the IoT environment and the underlying devices can be linked to. By ensuring this service, data collected inside the IoT system is available to other IoT systems. Still, the PCP monitors and secures these connections, enforcing the rules of access control defined at the moment of establishing connections with these “exterior” services. Being a policy enforcement point, the PCP permits the traffic flow, from end-point IoT devices, only to secure upper layer services (legitimate ECP entities).

By being a central hub where different gateways interconnect, the PCP has mechanisms of ensuring secure communication links between gateways and itself or between users and the services it is configured to offer. The security tokens needed to authenticate and authorize both users and gateways are manually provided by an administrator, in the management interface of the PCP.

Besides these active tasks that employ the use of the PCP in different scenarios of an IoT environment, the PCP is mostly a passive component. This is because the main idea behind our PCP is it to act as a repository integrating two main functionalities:

- a central point where anomaly detection data and graphs created by gateways can converge and offer an integrated overview of the IoT environment;
- a storage location in which valuable data (working data) gathered by the gateways can be pushed. This data is being stored in different areas, depending on the type of information it contains:
  ○ environmental data accessible to every requesting service;
  ○ historical data needed for reassessing the level of trust and reputation of each collector node or sensor;
  ○ privacy-aware data consisting of information related to an individual or his habits. This type of data has also assigned a certain tag, specific to each individual, as presented later in the paper.

**Figure 2:**
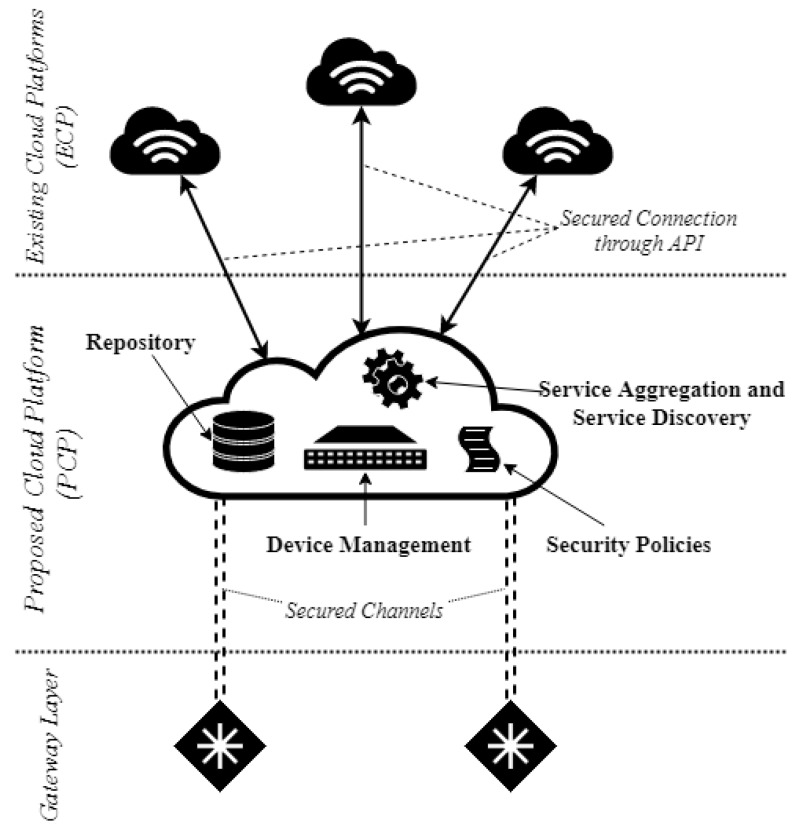
The architecture of the proposed Cloud platform.

This repository is a central database that stores information provided by gateways that also query and update this information based on their authorization levels. By querying this repository, gateways can correctly choose trusted links to enable connections between IoT devices from areas that are distinct, but connected in the same IoT system, as presented in Figure 2.

At this stage, the PCP is enabled only to store the above mentioned types of data and ensure secure access to it, without having the means of manipulating it. Still, if a certain degree of trust is ensured from the moment when the IoT environment is created and configured, the PCP could be extended to support methods of data integration and update, leading to the creation of a uniformed graph of anomalies detected across the entire IoT system. Even though this might bring an advantage in having an integrated overview of the devices’ anomaly detection characteristics, it implies also the use of the same or similar anomaly detection algorithms in each gateway, thus similar hardware configurations (easy to comply within a newly created IoT system, but harder in one that is updating an existing IoT environment).

### 3.2. Gateway Layer

Even though there are different models of IoT networks, gateway-centric is one of the most employed for health and social care applications. One of the main advantages brought by the gateway-centric model is that it consists of a central device that implements logic to coordinate the IoT sensors. From the cost perspective, having a gateway-centric network allows deploying a fleet of sensors with limited capabilities, given the fact that the gateway can be used to offload both security and data processing functions. The gateway is the element that links the end-point network segment with the upstream network elements, executes translation from lightweight to classic communication protocols or executes various security tasks like authentication, authorization and access control or packet filtering. The gateway can also act as a network access server on various layers: the gateway can execute a Layer 2 Extensible Authentication Protocol (EAP) authentication, a Message Queuing Telemetry Transport (MQTT) user/password based authentication or a custom authentication protocol transported as Constrained Application Protocol (CoAP) payload. IoT gateways do not have the same computational constraints as the end-point IoT devices, thus being suitable for offloading certain resource intensive tasks from the low-end devices, like security operations. Having a gateway-centric model also brings a series of advantages in the overall IoT network economics, by reducing the latency required to process IoT delivered data (real-time applications) and reducing the traffic between the sensors and the Cloud back-end.

Being a central IoT network element, gateways need adequate protection mechanisms against sophisticated DoS attacks in order to provide the network availability and to satisfy real-time constraints. Taking advantage of the IoT gateway capabilities, various architectures transform this device into a multi-tenant application hypervisor. For instance, the gateway can run an MQTT broker application or a CoAP client which aggregates data from multiple sensors.

The gateway plays a central role in our IoT security framework, having two main tasks: implementing a sensor anomaly detection module and a mechanism for an advanced network packet filtering. The previously mentioned IoT gateway tasks stress the necessity of implementing a security control plane that allows the end-point IoT devices to transmit and receive the sensor anomaly status and packet filtering related commands and data.

The architecture of the proposed security framework consists of a gateway and a suite of IoT end-point devices that enable security as a service structure. The security control plane between the end-point devices and the gateway consists of a publish-subscribe protocol, which allows an energy-efficient asynchronous communication path from the gateway to the nodes. The sensor anomaly detection control plane consists of the following commands: publish data and acquire the anomaly status. The gateway processes the anomaly detection requirement commands and executes the associated actions on the Cloud platform side. When the IoT nodes are acquiring data, the gateway parses the published information from the Cloud platform, it runs a sensor anomaly detection algorithm and delivers the status to the IoT devices. The sensor anomaly detection module has the following elements:

- The sub-module which is in charge of securing the communication between the gateway and the Cloud platform;
- The sub-module which secures the communication between the IoT end-nodes and the gateway;
- The sub-module which computes the IoT sensor anomaly status.

In this security scheme, the Cloud platform has the role of a sensor data repository, storing the IoT published data. The calculation of anomaly status is executed locally, on the gateway side, the Cloud module being a passive element which does not allow any data modification after the publish event. The proposed IoT security framework employs a sensor anomaly detection algorithm module with a well-defined interface. Thus, the security framework abstracts the actual anomaly algorithm implementation, offering only a plug-in module which allows running any type of anomaly detection algorithm. The security framework acts as a software container that injects in a custom anomaly detection algorithm implementation the input data collected from the Cloud platform. By adding a generic sensor anomaly detection algorithm support, the IoT security framework addresses the specific requirements of each deployment scenario.

Even though the IoT gateway is not a device with major resource constraints, the power consumption should be taken into consideration when running a sensor anomaly detection algorithm. In order to address this issue, we propose a Stochastic Petri Network (SPN) model of the gateway power consumption. This is an abstract model that indicates the number of times the gateway finite state machine enters a generic Process and Sleep state. In contrast to a finite state machine model which comprises states and conditions which must be satisfied to transition from one state into another, an SPN model consists of transitions, which are fired following a stochastic paradigm, and states (places) which hold the system resources. In our gateway model, the resources consist of requests received by the gateway (real time/critical requests and best effort requests which are held in the Queue, Real-time queue and Best-effort queue states) and execution resources (which are transitioned between the Process and Sleep states).

**Figure 3:**
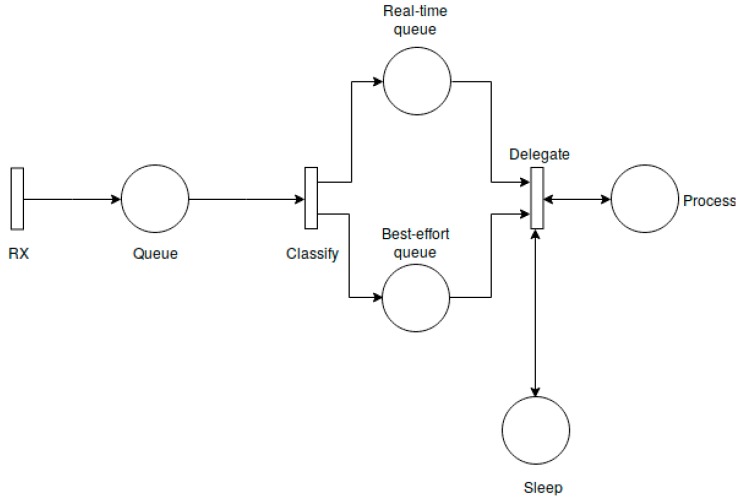
SPN power consumption model for sensor anomaly detection.

The SPN model has two main purposes: limiting the sensor anomaly computing tasks when the gateway has a low battery level and predicting the power consumption. More specifically, we propose an SPN API that allows a developer to translate a power consumption formal model into a software module that can be deployed on the gateway. As we have already shown in [23], an SPN model can be used to estimate the power consumption of an IoT gateway. By using the SPN model, the gateway administrator can make a prediction regarding the power consumption based on the network traffic characteristics (e.g., number of requests per time interval). This can help in planning the IoT network gateway provisioning and estimating maintenance operations. By having this planning, an administrator can further estimate the costs associated with running and maintaining security and network functions on edge IoT gateways. The proposed SPN power consumption model is a generic framework whose output depends on the actual anomaly detection algorithm and on the underlying hardware platform. An example of an SPN reputation power consumption model is depicted in Figure 3.

As it can be observed in our generic SPN model, we have the following transitions:

- *RX*—generates request messages for the gateway (using a random model).
- *Classify*—it classifies the messages based on their priority. Given the healthcare context, there can be two types of messages: contextual messages which are handled following a best-effort paradigm and critical messages which are handled on a real-time basis. This transition does not follow a stochastic model and it is triggered for every change occurred in the *Queue* states.
- *Delegate*—this transition has a hybrid behavior, having the next logic:
  ○ for every real-time message it executes a transition into the *Process* states (this behavior is not stochastic);
  ○ if there are no real-time messages and if the execution is currently in the *Process* states, then it executes a transition into the *Sleep* states with a probability given by Equation (1);
  ○ if there are no real-time messages and if the execution is currently in the *Sleep* states, then it executes another transition into the *Sleep* states, with a probability given by Equation (1);
  ○ if none of the previously mentioned steps are executed, this transition extracts a best-effort message and moves the execution into the *Process* states.

These transitions are accompanied by five SPN states as follows:

- *Queue*—accumulates both the critical and best effort requests;
- *Real-time queue*—accumulates the critical requests;
- *Best-effort queue*—accumulates the best-effort requests;
- *Sleep*—indicates the number of times the gateway transitions into a *Sleep* state;
- *Process*—indicates the number of times the gateway transitions into a *Process* state. This is the consumption intensive state which must be translated into a hardware dependent consumption metric.

Our SPN API allows introducing new states and transitions along with a custom transition frequency function. In our tests, we have used a battery level dependent function for the Delegate transition, as presented in Equation (1). This transition function uses as input both the gateway battery level along with a random variable and returns a boolean value which indicates if the SPN transition should be executed or not. If the Delegate transition is executed, the SPN machine moves into the Sleep states, thus, according to Equation (1), if the battery level is low, the probability of transitioning into the Sleep state increases:

delegate_func = f(battery_level, random_variable) = {True, random_variable > battery_level, False otherwise}(1)

**Figure 4:**
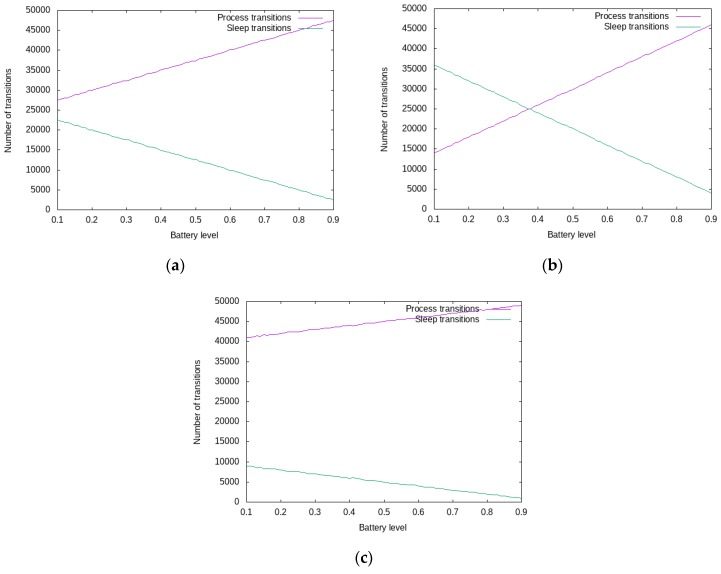
SPN consumption model of the IoT Security Gateway: (**a**) Uniform distributed real time/best effort messages; (**b**) Best effort predominant message; (**c**) Real time predominant messages.

Thus, a developer can model the power consumption of the IoT gateway in a custom manner and obtain the application specific trade-off between security and availability. In Figure 4 it can be observed that the number of transitions into the Sleep and Process states have a linear dependence on the battery level: the number of transitions into the Process state is increasing along with a high battery level, while the number of transitions into the Sleep state is increasing along with a low battery level.

Figure 4 consists of the following input data sets:

- uniform distribution of real-time and best-effort messages (Figure 4a);
- best-effort predominant messages which allow the system to increase the transitions into the *Sleep* states (Figure 4b);
- real-time predominant messages which force the system to increase the transitions into the *Process* states (Figure 4c).

The results from Figure 4 measure only the number of times an SPN transition is executed based on the input requests pattern. To further obtain actual power consumption metrics, the number of times the Process transition is executed must be correlated with the actual anomaly detection algorithm and hardware characteristics. One could obtain power consumption values by running the proposed SPN framework on an energy aware hardware emulator.

In an IoT network, the gateway executes a suite of communication processes, which cover the entire network stack, starting with data layer protocols and ending with the application layer protocols. The gateway role is augmented in a publish-subscribe network because the gateway can be the module that relays the packets between end-point IoT devices. In a client-server IoT network topology, the gateway translates packet from a lightweight protocol, bridging the upper layer Cloud network segment with the end-point network segment. Taking into consideration these characteristics, the gateway becomes a multi-tenant device which can execute various security processes (e.g., authentication, network access, policy enforcement), given its central role in an IoT network. One of the most important attacks in IoT networks is the Denial of Service (DoS) which can be split into two main categories:

- DoS on the end-point devices with the purpose of draining the battery and the availability of the device;
- DoS on the gateway side with the purpose of affecting the availability of the IoT network or services.

Our IoT security framework implements a packet filtering mechanism on the gateway side in order to mitigate DoS network attacks. The packet filtering module can cover all the networking stack layers, focusing on the Message Queuing Telemetry Transport (MQTT) / MQTT for Sensor Networks (MQTT-SN) and CoAP application layer protocols. The network filter layer consists of two sub-modules: the packet filter mechanism in the kernel side and the userspace filter component. The packet filtering on the kernel side is implemented using the Extended Berkeley Packet Filter (eBPF) technology. This technology allows deploying a lightweight network parser in the kernel virtual machine. The kernel network parser allows a rapid packet inspection which offers the possibility of dropping or modifying packets. This mechanism allows the authenticated and authorized IoT nodes to install custom filtering instructions in the network filter virtual machine on the gateway side. Thus, a low resource endpoint IoT device can offload the CPU intensive packet filtering task on the gateway side. By using this gateway delegated filtering mechanism, an IoT device can save CPU cycles for its’ own system and for the gateway system, dropping malicious network packets in the earliest possible stage. Such a dynamic security mechanism is suitable for mitigating MQTT attacks where a malicious publisher device sends DoS packets to a subscriber device.

The packet filtering architecture also uses an IoT node virtual instance on the gateway side, which keeps the device state (e.g., filtering commands, online state, etc.) and acts as a buffer between the gateway and the physical IoT device. Each packet filter routine uses a suite of packet qualifiers (e.g., source IP address, MQTT identifier, MQTT QoS value) which triggers the routine. A kernel space filtering mechanism requires the usage of an operating system (e.g., lightweight Linux), which can be a drawback for bare-metal systems or for systems that are not eBPF compatible. To overcome this drawback, the proposed IoT security framework implements a hybrid kernel/userspace filtering mechanism. The userspace filtering mechanism is implemented as an application protocol security module. The security module has a well-defined interface and it is called by the core packet application protocol daemon for each received packet. By using a packet filtering interface, the module can be easily replaced with a custom one without changing the core protocol implementation. The userspace filtering module consists of a collection of routines that can execute tasks like packet inspection or accept/drop the packet based on the authentication/authorization state. Like the kernel filtering mechanism, the userspace module uses packet qualifiers that can lead to a packet reject action if they are correlated with the sender/receiver authentication state. At the gateway side, the userspace filtering module can receive filtering policies (ACL structure) from the endpoint nodes by using the secure control communication channel. In a particular MQTT scenario, an endpoint IoT node can install the following policies:

- Packet rate limit which is correlated with the received (IoT node) battery level;
- Asynchronous authentication of the publisher IoT device (the policy is installed by the subscriber IoT device which wants to receive data only from trusted nodes);
- Asynchronous authorization of the publisher IoT device;
- Blacklisting nodes based on the protocol URI (e.g., MQTT client id);
- Packet dropping based on different qualifies (e.g., header values, payload value).

**Figure 5:**
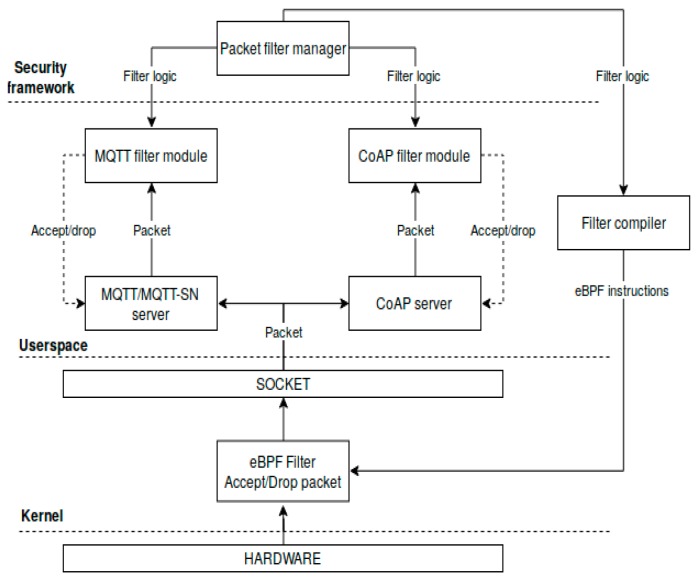
The architecture of the gateway packet filtering module.

Regarding the CoAP protocol, the gateway filtering solution handles the CoAP proxy scenario. Thus, the IoT framework permits installing security policies which authenticate both the CoAP server and client and limits the rate of CoAP responses based on the client’s battery level. The CoAP rate limiting capability addresses the multicast responses which can be easily used to execute a battery drain attack. The gateway packet filtering architecture is depicted in Figure 5.

The userspace and the kernel filtering module are not mutually exclusive. Taking into consideration the limitations of in-kernel eBPF network parsers (e.g., loop restrictions), our hybrid packet inspection solution is a multi-stage filtering solution. Thus, the complex part of the filtering routine can be installed on the userspace module (e.g., authentication/authorization verification or querying other modules when validating the packet). The simplest part of the ACL can be installed in the kernel filtering module, which achieves higher speeds and optimizations in the packet rejection process.

Our multi-stage filtering mechanism also addresses the IoT TLS/DTLS encrypted traffic. The in-kernel filtering module does not have the TLS/DTLS session key, thus it cannot inspect encrypted traffic payload. This scenario is addressed by the userspace filtering module which is attached to the application protocol process (e.g., MQTT broker process) and receives plain-text packets that can be inspected normally (with the associated performance penalties of userspace filtering).

Both the userspace and kernel security modules employ a cooperative filtering paradigm between the endpoint IoT nodes and the gateway, which addresses the dynamic character of IoT networks.

The software security module which runs on the gateway has a lightweight structure and it does not rely on the underlying hardware capabilities, being suitable even for battery powered devices and for applications that cannot benefit from the gateway-centric model. Taking this into consideration, the security functions deployed on the gateway can be executed on regular IoT nodes, thus addressing other IoT network topologies than the gateway-centric ones. For instance, these security functions could be executed by specially designated IoT nodes from a mesh or multi-hop routing topology.

### 3.3. IoT Device Layer

The outstanding feature of IoT devices is their ability to ensure system security using limited resources. The endpoints are the most vulnerable to attacks because they are often installed in open space environments and an attacker can gain physical access to them, leading to security breaches in which the attacker can compromise the hardware and software configurations and alter the information transmitted over the network. In this regard, the proposed architecture includes, at the device layer, a remote attestation framework that has the role of checking the hardware and software configuration.

Attestation is a mechanism for the gateway to measure the IoT end-point integrity and remove the node from the network if compromised. Traditionally, remote attestation is achieved by means of secure hardware, like Trusted Platform Module (TPM) incurring resource and monetary costs which are not acceptable for the majority of IoT devices. Attestation can also be achieved using a software-only solution, but this mechanism may not reach the desired security level of a particular IoT application. There are also hybrid solutions that combine the previously mentioned mechanisms in order to obtain a trade-off between the security level and the costs (including hardware complexity).

There are a series of papers that propose different types of attestation solutions that can fall into one of the three categories presented before. Since hardware-based remote attestation has a greater cost than the remaining two categories, we will focus mainly on software or hybrid solutions. In [24] authors have proposed a software-based technique that takes advantage of specially designed functions that will have a different way of executing if an attacker tries to modify it, introducing extra delays, therefore any alteration of the base software image will be detected and reported. Following the hybrid approach, in [25] the authors proposed the Secure and Minimal Architecture for (establishing a dynamic) Root of Trust (SMART), through which they use the properties of the Read-Only Memory (ROM) module found in majority of low-end microcontroller unit (MCU) architectures to replicate a few of the features that a TPM has (e.g., secure key storage area, reserved zone for attestation code or access control for enforcing the protection of specially defined areas). Another proposal is given in [26] and consists of a technique that uses Physically Unclonable Functions (PUF) to detect any variation in the flow of function execution, by benefiting of side-effects in the processor manufacturing to generate unique hardware outputs for each input. For IoT devices that are running a lightweight Linux operating system, there are also mechanisms like Integrity Measurement Architecture (IMA) that can attest the system run-time integrity. Linux IMA can also be integrated with a TPM in order to compare the run-time integrity value with a data source that cannot be modified.

**Figure 6:**
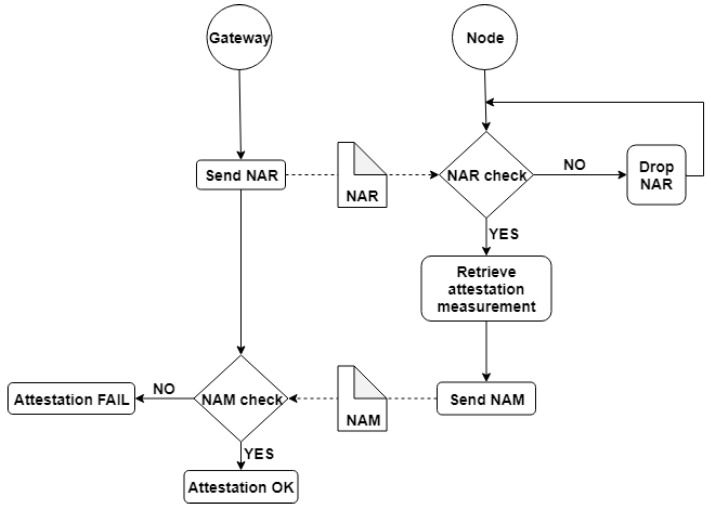
The architecture of the Remote Node Attestation Module.

The proposed attestation framework has a generic structure that implements a secure message protocol and relies on an external module for obtaining the integrity measurements (e.g., TPM, Linux IMA). Whenever the gateway wants to check the integrity of a node, it sends a Node Attestation Request (NAR) message to the IoT device. The NAR message consists of a nonce and an Hash-based Message Authentication Code (HMAC) signature, computed over the nonce value, using a pre-shared key established between the endpoint device and the gateway during the device enrollment phase. After receiving the NAR message, the node validates it by verifying the HMAC signature, thus mitigating DoS attacks where a malicious entity sends multiple attestation requests to the device in order to exhaust the computing/energy resources of the node. If the NAR message is validated, the endpoint device executes the attestation measurements by means of an external module (e.g., methods mentioned at the beginning of this subsection). After obtaining the integrity measurement values, the endpoint device sends to the gateway a Node Attestation Measurement (NAM) message which consists of integrity measurement values along with an HMAC signature, computed over the integrity measurement values concatenated with the nonce received from the gateway. The HMAC signature of the NAM message is computed using the same pre-shared private key, established between the IoT device and the gateway. The gateway validates the HMAC signature of the NAM packet (using the request nonce and the pre-shared key) and checks if the integrity measurement values are the same with the values stored on the gateway side. If the signature is invalid or the integrity measurement values are incorrect, the gateway marks the device as compromised and ceases any communication with it. The attestation state machine on the IoT device and gateway side take into consideration a connectionless transport protocol, sending the messages multiple times before transitioning into an error state. The remote attestation message protocol is presented in Figure 6.

Through the dynamic approach of the gateway to node profiling and suspicious behavior detection, devices are continuously monitored and in case a device seems to have been compromised, then the gateway can request the starting of the attestation cycle. Abnormal behavior of the nodes can be detected by inbound and outbound traffic analysis. A node that no longer performs its tasks or starts making forbidden connections can be suspected that is under the direct control of an attacker. In this case, the gateway sends to the node a verification request and the latter starts the attestation cycle.

Regarding the security of the communication channel, messages between IoT devices and gateway may be protected using an Integrity Check Value (ICV), which consists of an HMAC computed over the message payload. The ICV field usage allows detection of packet tampering and replay attacks, which could allow an attacker to manipulate the sensor aggregated information. The HMAC algorithm employs the aforementioned pre-shared key between the endpoint device and the gateway.

The proposed security framework also takes into consideration the privacy required by specific user-related data. If data is mainly collected through a group of body sensors interconnected in a BAN that has a specific sink through which data is exported to gateways, we can consider that sink as a collector node. For enforcing the anonymization of data being sent to gateways and upper levels in the framework, the collector node is initialized for each new individual with a different tag that will be associated with all data collected and sent by that node. This tag will be generated based on the person’s identity features (social security number, name, and age) that will be fed as input to a hash function, to ensure the randomness of the tag. By creating the tag in this manner, any future data associated with a person can be processed without employing the user’s identity. Also, through the tag, we create a decoupling of data and a person’s identity, without affecting any of the functionality offered by the IoT system.

Considering the limited resources that an IoT device has, it is very important to carefully monitor them, because an overload of a node can lead to lower computational performance or inactivity. In the proposed architecture, every node implements a module in charge of monitoring the load level in order to optimize the use of resources such as memory loading, CPU usage or battery level. Any exceeding of acceptable performance thresholds that can slow down the system will be managed at the gateway layer by adapting traffic and activities in order to improve the distribution of workloads across the system elements.

**Figure 7:**
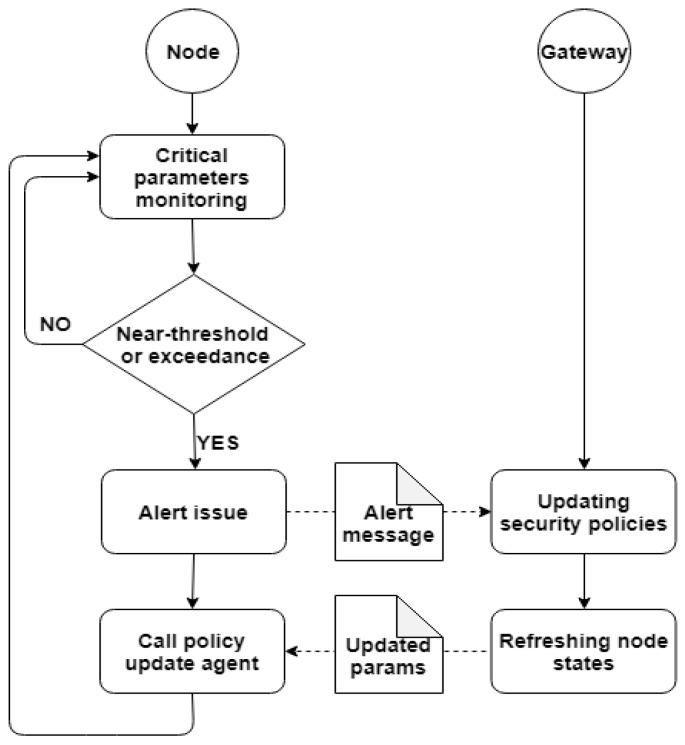
The architecture of the Node Status Monitoring Module.

When the gateway receives an alert from the node about its status, it will update the security policies and forward the information to the node, which in turn will call the policy update agent installed at its level. The policy update agent can also be called by the node when it detects that the preset thresholds are exceeded. In this case, the node initiates the process of updating security policies by sending a request to the gateway (see Figure 7).

### 3.4. Management Layer

In order to offer a complete security framework architecture, for the user level we have proposed a web application as a management tool. The web application allows the security administrator, through the graphical interface, to enable packet filtering policies or to create a blacklist with devices that are not taken into account when the anomaly detection score is computed. Regarding the sensor anomaly detection process, the security administrator can set certain parameters for monitoring the process and alerting him whenever a triggered parameter is activated, for example when the maximum value is reached, then the data is transmitted to the web application and the administrator can analyze it and take action.

The web application can be also considered the logging center of the IoT system. The logs from the nodes reach the gateway which will redirect them to the web platform, thus the logs will be available for the security administrator. In order to address the incompatibility issues with existing proprietary solutions, the web platform interacts with gateways using Representational State Transfer (REST) calls secured by the TLS protocol.

Through the web application, the security administrator can monitor and keep track of packet streams, having the possibility to view the rate at which packages are processed. The packet processing rate allows the security administrator to evaluate, in real time, the effectiveness of the installed filtering policies and to observe the pattern of a DoS attack.

## 4. Testing and Results Analysis

In realizing the proof-of-concept for different modules of our proposed security framework, we used open-source components that offered a proven solution at a specific layer of the architecture while enabling various customizations needed to implement the functionality described in the previous section. For managing connections and enforcing the monitoring and access control features of the Cloud module, we integrated a Zuul proxy solution, through which we can implement security rules. Acting as a service aggregator, the Zuul proxy empowered the use of filtering mechanisms on packets and the implementation of the service discovery mechanism described in the previous section. The passive component of the Cloud module, namely the ambient environment repository, needs to store information in a structure similar to a graph. Considering this, we chose to implement the repository using a Not Only Structured Query Language (NoSQL) solution and picked MongoDB for this purpose, given its maturity and the availability of drivers for several programming languages, such as C++ and Java.

For the core gateway security framework implementation we used Eclipse Kura which interacts with other local systems (e.g., Mosquitto MQTT broker) by means of IPC mechanisms. Thus, our reputation and SPN consumption modules are deployed as Kura OSGi containers. For the Cloud interaction, we used the regular Java Jetty HyperText Transfer Protocol (HTTP) client for consuming REST services exposed by the Cloud module: sensing data publishing, sensing data retrieval. Regarding the communication stack, we used separate IoT protocol daemons like Mosquitto for MQTT broker, Eclipse Paho for MQTT-SN and Eclipse Californium for the CoAP server.

As stressed in the previous section, packet filtering is a complex task that cannot be executed by resource constrained endpoint IoT devices. This process needs continuous optimizations even on the gateway side in order to reduce security allocated resources (CPU cycles, memory). Given that IoT network applications require low-latency communications, a DoS attack could be executed even if the gateway allocates resources for dropping the malicious packets, thus being unable to dispatch the legitimate packets in the required time frame. In order to evaluate the packet filtering module for our gateway side IoT framework, we tested a scenario where an attacker executes a DoS using the MQTT-SN protocol. IoT gateways are usually low-cost devices deployed at the edge of the network. These devices are not equipped with advanced hardware based filtering modules like Application-Specific Integrated Circuits (ASIC), Field Programmable Gate Arrays (FPGA) or Intelligent Network Interface Controllers (SmartNIC). Considering these characteristics, the gateway filtering solutions must be executed exclusively in software, either in the kernel or in userspace. Kernel filtering solutions bring the advantage of dropping a malicious packet in the earliest stage of the network data processing pipeline. Thus, a kernel based early packet drop mechanism saves resources because kernel packet (e.g., Linux skb structure) data structures will not be allocated and the receiver process will not be scheduled, thus avoiding important context switch penalties. A kernel packet filter module brings the drawback of complex development and the security risks of compromising or crashing the system when deploying an untrusted kernel module. The userspace filtering solutions bring more flexibility in terms of packet processing with the penalties of costly operating system context switches. In the case of DoS attacks, the system is delayed by executing multiple context switches, being unable to process packets in the required time frame.

**Figure 8:**
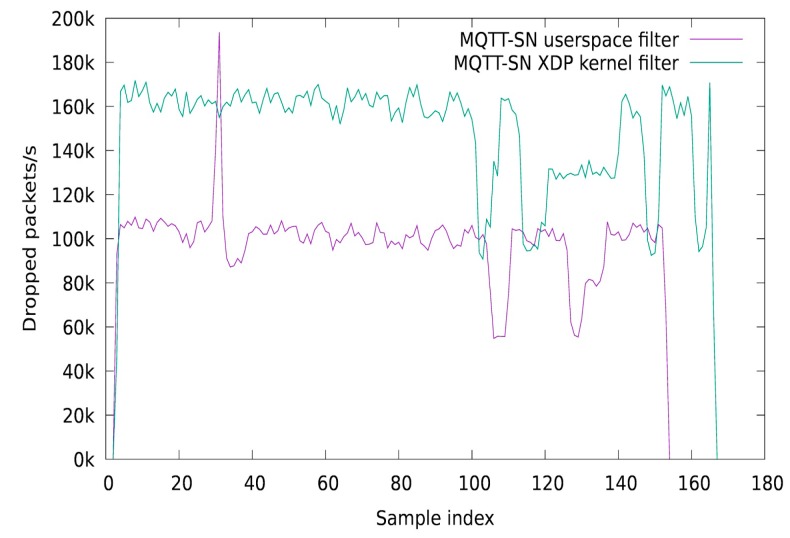
Drop packet rate (per second), in a DoS attack scenario.

A typical MQTT-SN scenario consists of IoT sensors that transmit information to a central broker, using MQTT-SN PUBLISH, via a transparent or aggregator gateway. In our experiments, we simulated an MQTT-SN PUBLISH DoS attack, where a malicious device tries to allocate the gateway filtering resources in order to block the legitimate packet processing. The implementation consists of a dynamic kernel based MQTT-SN filtering solution, using the eBPF technology. More specifically, we used the eXpress Data Path (XDP) / Iovisor open-source project which allows programming the Linux kernel network data path using eBPF instructions generated from C programs (using the clang compiler). Our implementation uses the BPF Compiler Collection (BCC) framework, which allows injecting the in-kernel eBPF instructions using a python front-end. For keeping a session on the kernel filter module and for collecting statistics, we used eBPF maps to communicate between the controller and the filter module. For the MQTT-SN gateway, we used the Eclipse Paho MQTT-SN project, which was integrated with our userspace ACL module. We simulated an attack scenario where one or more IoT devices are compromised and used by the attack coordinator to execute DoS actions on the gateway side, by sending a continuous flow of MQTT-SN PUBLISH packets. For the client (attacker) which floods the network with MQTT-SN PUBLISH packets, we also used the Eclipse Paho libraries. In a first experiment, we measured the number of dropped packets per second, by using the XDP kernel filter and the userspace module. For obtaining the kernel packet drop statistics, we used an eBPF array to store the values and a Python script which polls the module every second. For our testing scenario, we dropped MQTT-SN packets based on the QoS value, a protocol feature that can be easily exploited by an attacker. In Figure 8 is presented the MQTT-SN drop rate per second, when using a kernel XDP packet filter compared with a userspace packet filter. As it can be observed, the traffic drop process follows the same pattern, with the XDP kernel drop rate being 60k packet higher than the userspace alternative. The results for this test case were obtained after the attacker device sent 100 million MQTT-SN packets.

**Figure 9:**
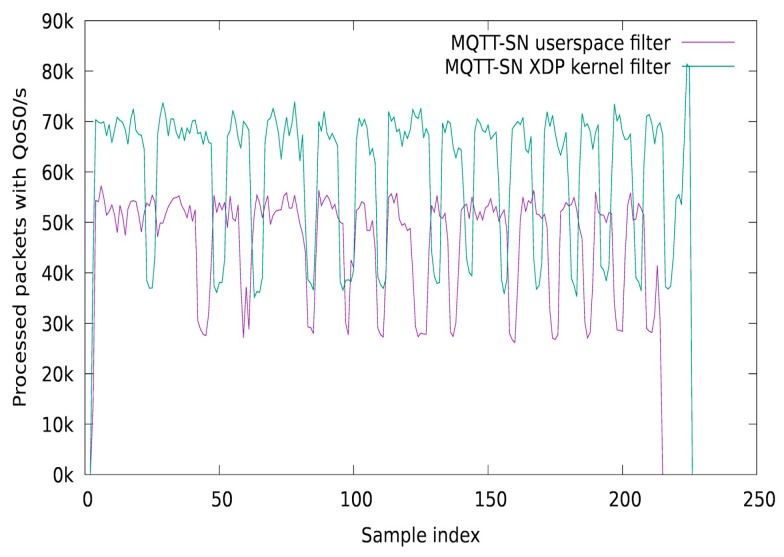
Processed packet rate (per second) in a DoS attack scenario.

In Figure 9 are presented the results of a second test case, where we analyze the rate of processed packets per second in a case of DoS attack. The DoS attack scenario consists of a malicious device that sends 100 million MQTT-SN packets with a QoS value of 1 and a legitimate device that sends the same number of packets with a QoS value of 0. As it can be observed, when the gateway uses the XDP kernel filtering method, it can process 20 k more legitimate packets, than when using the userspace alternative.

As noted in Section 3.3, before starting any data transfer, the gateway executes a remote attestation protocol with the IoT device. To validate the gateway attestation state machine, we simulated a compromised IoT device by tampering the attestation response packet and by delivering a wrong integrity measurement hash.

The DoS attack experimental results show that our proposed method drastically improves the packet drop rate, without requiring any dedicated hardware. Such a solution permits deploying off-the-shelf low cost gateways that can handle the security and network functions for the associated IoT network. This brings flexibility in choosing the hardware equipment to be installed on the IoT network edge. Also, by removing the necessity of having dedicated hardware for packet filtering (e.g., ASICs), the power consumption on the gateway side is significantly reduced. Implementing the security filtering functions in software also brings flexibility in terms of software updates, taking into consideration the continuously changing IoT attack techniques. In contrast, while ASIC filtering mechanism can provide a better performance in dropping packets, changing a hardware generation brings increased costs and delays, which can be unacceptable for the agility of IoT markets.

## 5. Conclusions and Future Work

IoT development is a complex task, due to the multitude of elements which compose an IoT system, thus a standard framework accelerates considerably the time to market of an IoT product. Moreover, the importance of an IoT security framework is augmented in the current context, taking into consideration that IoT devices operate with user private data and that mature IoT security solutions can increase the IoT technology adoption. Our security framework targets all the layers of an IoT application: end-point network, gateway/edge, Cloud and management, trying to offer an all-in-one solution for designing an IoT security system.

The proposed security framework was designed to be deployed on low-cost commodity IoT hardware. It does not rely on dedicated hardware for executing security and network functions offload, trying to meet a trade-off between performance and costs, as presented in the experimental results section. The experimental results analyze both functional aspects (power consumption model) and security aspects (DoS attacks mitigation mechanism) in order to validate one of the main characteristics of our system: a high availability security platform. This characteristic is critical for ambient intelligence applications that need to rely on a highly available infrastructure that must provide security functions. Costs and flexibility are also two important characteristics of an ambient intelligence application infrastructure, which are addressed by our system, taking into consideration that the security and network edge devices have high costs in terms of acquisition/deployment and maintenance. Also, given the fact that our solution is implemented using software-only functions, the system can be easily updated in order to meet the dynamic of the IoT security landscape, where new attacks and vulnerabilities are discovered frequently.

Concerning the security attacks, based on the experimental results, we can conclude that our system is resilient to DoS attacks and to energy exhaustion attacks. The simulated DoS attack consists of one or more compromised IoT devices that send malicious network packets to the gateway, this attack being handled at the gateway side by dropping packets in an optimized manner while serving normal requests. The energy exhaustion attack is also handled at the gateway side by the SPN power consumption model which delays, in a stochastic manner, the execution of the anomaly detection algorithm when the gateway’s battery is low.

Regarding our future work, we plan to integrate and test various reputation algorithms to offer a suite of default algorithms which can address various IoT interactions model, like Barabasi-Albert, Watts-Strogatz or random. Another research direction is extending our security framework with a Linux Containers (LXC) manager in order to allow endpoint IoT devices to run complex filtering applications on the gateway side. Taking into consideration that a Trusted Execution Environment (TEE) is important in the IoT context, mainly because of the deployment scenario (e.g., low physical protection, third-party software), we also plan to add a TEE compatible abstract software module. The TEE aware module will allow our IoT security framework to be integrated with TEE hardware and as a default implementation, we plan to use OpenTEE. Regarding our packet filter solution, we plan to integrate our security framework with an eBPF aware SmartNIC which can offload the gateway CPU from the filtering process while providing the same flexibility as our in-kernel packet protection mechanism.

From the anomaly detection point of view, we plan to implement and test an algorithm based on the prediction of node value and afterward use the predicted value to detect potential data anomalies. Our goal is to implement a Sequential Minimal Optimization (SMO) Regression, which is the most suitable prediction method for healthcare [4], and to catch the error by comparing the values with a dynamic threshold. A dynamic value for the threshold is efficient in healthcare systems because of tracked parameters which may vary depending on the activity that the person undertakes. The threshold will be determined using the sliding window algorithm.

In this paper, we presented a modular IoT security framework for ambient intelligence environment which addresses all the IoT application layers and targets various security areas. This framework aims to be platform independent and it can be integrated with third-party IoT platforms, separating the security tasks from the functional processes.
